# Draft Genome Sequence of Arthrobacter oryzae TNBS02, a Bacterium Containing Heavy Metal Resistance Genes, Isolated from Soil of Antarctica

**DOI:** 10.1128/MRA.01501-18

**Published:** 2019-01-24

**Authors:** Yong-Joon Cho, Ahnna Cho, Soon Gyu Hong, Han-Gu Choi, Ok-Sun Kim

**Affiliations:** a Korea Polar Research Institute, Incheon, Republic of Korea; University of Maryland School of Medicine

## Abstract

Arthrobacter oryzae TNBS02 was isolated from soil at Terra Nova Bay of Victoria Land, Antarctica. The genome consists of a chromosome with 4,248,670 bp which contains a total of 3,994 genes. It was identified that this strain has many heavy metal resistance genes similar to other *Arthrobacter* strains isolated from contaminated environments.

## Abstract (precis)

Arthrobacter oryzae TNBS02 was isolated from soil at Terra Nova Bay of Victoria Land, Antarctica. The genome consists of a chromosome with 4,248,670 bp which contains a total of 3,994 genes.

## ANNOUNCEMENT

Arthrobacter oryzae TNBS02, Gram-positive obligate aerobe, was isolated from soil at Terra Nova Bay of Victoria Land, Antarctica. The coordinates of the sampling site were 74°37′26.0″S, 164°13′49.2″E, and this sample was collected on February 3, 2011 to find out what kind of microorganisms are present in the natural Antarctic environment before the Jang Bogo Station, South Korean research station, was built on the site. The sediment sample was diluted in phosphate-buffered saline (PBS) buffer and incubated in R2A medium at 10°C for 2 weeks. This strain showed 99.71% similarity with A. oryzae KV-651 type strain (1) by 16S rRNA gene typing using universal 27F-1492R primers and the EzTaxon-e database (2).

The genomic DNA of strain TNBS02 was extracted from a culture in R2A liquid medium using a PowerSoil DNA isolation kit (Qiagen, USA). The concentration and purity were determined with a Qubit 2.0 fluorometer (Invitrogen, USA), and 1 µg of genomic DNA was passed to the next step. A Nextera DNA flex library prep kit (catalog number 20018704; Illumina, USA) was used to make the DNA library for next-generation sequencing (NGS) following the manufacturer’s protocol. The sequencing was run on an Illumina MiSeq instrument with 300-bp paired-end cycles and produced 2,610,967 paired-end reads.

Raw data were cleaned with Trimmomatic v.0.36 (3) with default parameters for removing adapters and quality trimming. The assembly was carried out using resulting reads by SPAdes v.3.1.2 (4) with default parameters. The final assembly yielded 158 contigs with a length 4,248,670 bp; the *N*_50_ contig length was 92,154 bp, the GC content was 65.47%, and genome coverage was 184.97×. Gene prediction and annotation were carried out using the NCBI Prokaryotic Genome Annotation Pipeline (5). Also, BlastKOALA (6) against the “species_prokaryotes” database was used for functional annotation and KEGG pathway mapping. Finally, a total of 3,994 genes, including 3,771 coding genes, 5 rRNAs, 50 tRNAs, 3 noncoding RNAs (ncRNAs), 165 pseudogenes, and 3 CRISPR arrays were identified.

Several *Arthrobacter* strains were isolated from the environment contaminated with chemicals and metals and often had high resistance to heavy metals (7, 8). Surprisingly, the genome of A. oryzae TNBS02 contains many genes related to resistance to heavy metals, despite being isolated from the Antarctica that is unaffected by human activity. At first, *arsB* and *arsC* (locus_tag D7003_06960 and D7003_06965, respectively), the genes related to arsenate resistance, were identified, but the expected transcriptional regulator was not found, suggesting that there are other mechanisms to regulate this regulon. Also, the genome has genes for four tellurium resistance proteins (D7003_17165 to D7003_17175 and D7003_17190), two nickel-transporting operons (D7003_02150 to D7003_02165 and D7003_10900 to D7003_10915), copper resistance protein CopA (D7003_02310), a molybdate transport operon (D7003_01615 to D7003_01625), and heavy metal ion transporter CscZ (D7003_17260) related to metal resistance.

We have found that this genome has various heavy metal resistance genes despite that the natural environment is without pollution. The information of this genome may provide insight into the genetic basis for heavy metal resistance of bacteria in comparison with the strains found in other contaminated environments.

### Data availability.

Raw Illumina sequence reads were deposited in the Sequence Read Archive (SRA) under the accession number PRJNA503312, and the genome sequences and annotations were deposited in GenBank under the accession number RBED01000000.
